# Depressive symptoms in youth with ADHD: the role of impairments in cognitive emotion regulation

**DOI:** 10.1007/s00406-022-01382-z

**Published:** 2022-02-02

**Authors:** Jutta S. Mayer, Geva A. Brandt, Juliane Medda, Ulrike Basten, Oliver Grimm, Andreas Reif, Christine M. Freitag

**Affiliations:** 1 Department of Child and Adolescent Psychiatry, Psychosomatics and Psychotherapy, University Hospital Frankfurt, Goethe University, Deutschordenstraße 50, 60528 Frankfurt am Main, Germany; 2 Department of Psychiatry and Psychotherapy, Central Institute of Mental Health, Medical Faculty Mannheim, Heidelberg University, J 5, 68159 Mannheim, Germany; 3 Department of Psychology, University of Koblenz-Landau, Fortstraße 7, 76829 Landau in der Pfalz, Germany; 4 Department of Psychiatry, Psychosomatic Medicine and Psychotherapy, University Hospital Frankfurt, Heinrich-Hoffmann-Str. 10, 60528 Frankfurt am Main, Germany

**Keywords:** Attention-deficit/hyperactivity disorder, ADHD, Depression, Major depressive disorder, Comorbidity, Emotion regulation, Implicit, Explicit, Cognitive

## Abstract

Youth with attention-deficit/hyperactivity disorder (ADHD) are at increased risk to develop co-morbid depression. Identifying factors that contribute to depression risk may allow early intervention and prevention. Poor emotion regulation, which is common in adolescents, is a candidate risk factor. Impaired cognitive emotion regulation is a fundamental characteristic of depression and depression risk in the general population. However, little is known about cognitive emotion regulation in youth with ADHD and its link to depression and depression risk. Using explicit and implicit measures, this study assessed cognitive emotion regulation in youth with ADHD (*N* = 40) compared to demographically matched healthy controls (*N* = 40) and determined the association with depressive symptomatology. As explicit measure, we assessed the use of cognitive emotion regulation strategies via self-report. As implicit measure, performance in an ambiguous cue-conditioning task was assessed as indicator of affective bias in the processing of information. Compared to controls, patients reported more frequent use of maladaptive (i.e., self-blame, catastrophizing, and rumination) and less frequent use of adaptive (i.e., positive reappraisal) emotion regulation strategies. This pattern was associated with the severity of current depressive symptoms in patients. In the implicit measure of cognitive bias, there was no significant difference in response of patients and controls and no association with depression. Our findings point to depression-related alterations in the use of cognitive emotion regulation strategies in youth with ADHD. The study suggests those alterations as a candidate risk factor for ADHD-depression comorbidity that may be used for risk assessment and prevention strategies.

**Supplementary Information:**

The online version contains supplementary material available at 10.1007/s00406-022-01382-z.

## Introduction

Attention-deficit/hyperactivity disorder (ADHD) is a neurodevelopmental condition defined by a persistent and cross-situational pattern of age-inappropriate inattention and/or hyperactivity-impulsivity that leads to functional impairment [1]. Being a prevalent neurodevelopmental disorder with childhood onset, ADHD is also often the entry point into a trajectory defined by a high risk for co-morbid psychiatric disorders [2–5]. Mood disorders such as Major Depressive Disorder are among the most common comorbidities in adulthood [6–8] with prevalence rates considerably increasing when patients transition from childhood into adulthood [2–5, 9, 10]. Longitudinal studies suggest that children and adolescents with ADHD are at increased risk of developing depression when they reach adulthood [4, 11–14]. The co-occurrence of depression significantly worsens health outcomes (including the risk for completing suicide), causes psychosocial impairment and lower quality of life, and increases medical costs compared to those resulting from either disorder alone [9, 14–16]. Therefore, a better understanding of the factors [13, 17] that contribute to the increased risk for depression among patients with ADHD during the particular sensitive phase of adolescence and young adulthood is needed and would provide new opportunities in the development of early intervention and prevention strategies.

Psychological factors associated with ADHD and depression may mediate the pathway(s) from ADHD to depression [13, 17]. Poor emotion regulation, defined as an individual’s ability to modify an emotional state so as to promote adaptive, goal-oriented behaviours has been suggested as an important intermediate psychological risk factor for ADHD-depression comorbidity during adolescence [18–21]. However, emotion regulation is a broad psychological construct [22], and research is needed to clarify which components of emotion regulation are altered in youth with ADHD [23–26] and are associated with co-morbid depression or depression risk.

According to the temporal model of emotion regulation [27, 28], the ability to select, attend to, and appraise emotionally arousing stimuli determines the initial experience of an emotional state and the individual’s emotional reactivity. Emotional reactivity, also termed emotional impulsivity in the context of ADHD, refers to an individual’s threshold, intensity, and duration of affective arousal. Subsequently, modulation efforts take place that comprise automatic processes at multiple levels of processing as well as regulatory strategies, in order to promote adaptive responses to the emotional state or reactivity. It is well known that patients with ADHD show increased levels of emotional impulsivity, defined as including low frustration tolerance, quickness to anger, irritability, and emotional excitability, indicating poor emotion regulation in this group of patients especially during adolescence and early adulthood [25, 26]. While emotional impulsivity is neglected in current diagnostic criteria of ADHD, its fundamental role [25, 29] also clearly manifests at the genetic level [30]. Importantly, irritability has been associated with co-morbid depressive symptoms and depression risk in children with ADHD as shown in cross-sectional and longitudinal studies [18, 20, 31]. In addition, some evidence suggests that youth with ADHD also have difficulties in effectively modulating the intensity of inappropriate emotions in response to the initial emotional experience, and generating and maintaining appropriate emotions [24, 26]. However, it is not known to what degree alterations in these regulatory abilities are associated with co-morbid depressive symptomatology and depression risk.

To address this issue, the current study focused on cognitive emotion regulation, which is largely unknown in patients with ADHD but constitutes a key determinant of the capacity to regulate negative emotions in depression and has been associated with depression risk in the general population [32–38]. Cognitive emotion regulation can be studied via explicit measures such as direct self-reports that reveal information about the subjective experience and real world manifestations of cognitive emotion regulation. Furthermore, implicit measures that use behaviour in an experimental situation as an indicator of cognitive emotion regulation can be used [23, 27]. As indicated by self-reports, depressed individuals use maladaptive cognitive emotion regulation strategies (e.g., rumination and avoidance) more often, and adaptive strategies (e.g., reappraisal, acceptance) less often than non-affected individuals [37, 39–42]. The ruminative response style has been consistently observed even after remission [40, 43, 44], and is an important risk factor for depression onset [45]. Furthermore, as indicated by performance in cognitive tasks, individuals with depression show preferential processing of negative/mood-congruent information across multiple forms of cognition including attention [46, 47], memory [48], and the interpretation of ambiguous information [36, 49, 50]. These mood-congruent cognitive biases may affect people’s ability to regulate affect thereby providing the basis for an increased vulnerability to depression [37]. Consistent with this assumption, negative biases in the processing of information have been associated with the maintenance and the development of depression [33–36, 50–56].

There is little research on cognitive emotion regulation in patients with ADHD specifically during the potentially vulnerable phase of adolescence and early adulthood [26]. Preliminary findings in adults with ADHD point to the more frequent use of maladaptive emotion regulation strategies [57] which may be associated with lifetime depression [58]. Dysfunctional attitudes, negative attributional styles, and automatic thoughts have also been reported in adolescents and adults with ADHD, however, it is not clear to what degree these cognitive processes reflect current depressive symptomatology or risk for lifetime depression [58–62]. Few studies assessed negativity biases in ADHD and findings are mixed. Some studies in youth with ADHD revealed a negative attentional bias [63–65], but co-morbid depressive symptoms were not taken into account. One study reported that greater attention away from negative emotional information was related to higher levels of depression in adolescents with ADHD [62]. A less positive memory bias has been associated with externalizing problems in adolescents with ADHD [66]. An investigation of interpretation of ambiguous information in adults with ADHD in an interpersonal context did not find evidence for a negativity bias [67].

The goal of this study was to better understand cognitive emotion regulation in ADHD and its role in the development of co-morbid depression during the critical developmental window of adolescence and early adulthood. To this end, we assessed cognitive emotion regulation - previously associated with depression risk - in adolescents and young adults with ADHD (14–34 years) compared to demographically matched healthy controls (HC) and determined the association with current depressive symptomatology. The study compared effects for an explicit and an implicit measure of cognitive emotion regulation. As explicit measure, we assessed the use of adaptive and maladaptive cognitive emotion regulation strategies in daily life via direct self-report using the Cognitive Emotion Regulation Questionnaire (CERQ). As implicit measure, we assessed performance in an ambiguous cue-conditioning task as a behavioural indicator of cognitive bias. Current depressive symptoms and diagnoses were determined by validated self-reports, clinician-based symptom ratings, and diagnostic interviews.

We reasoned that if deficits in cognitive emotion regulation conferred risk for ADHD-depression comorbidity, youth with ADHD would use maladaptive cognitive emotion regulation strategies more often and adaptive strategies less often than HC as assessed with the explicit measure. We also expected to find a stronger bias towards negative interpretations of ambiguous information in patients compared to HC as assessed with the implicit measure. Furthermore, if poor cognitive emotion regulation was closely related to ADHD-depression comorbidity, we would expect alterations in cognitive emotion regulation as assessed with the explicit and the implicit measure to be associated with current depressive symptoms in our sample of youth with ADHD.

## Methods

### Participants

Forty adolescents and young adults diagnosed with ADHD (mean age: 22.93 years, *SD* = 5.60) and 40 demographically matched HC (mean age: 20.80 years, *SD* = 5.29) participated in the study (Table 1). Patients were recruited from the University Hospital Frankfurt. Additional patients and HC were recruited from the community. The data reported in this study was collected at baseline within a larger intervention study [68].

**Table 1:** Demographic and clinical information

	ADHD *N* = 40	HC *N* = 40	Test statistic
Age	22.93 (5.60)	20.80 (5.29)	*t*(78) = 1.75, *p* = 0.09
Age range	14-34	14-34	
Adolescents/adults, *N*	9/31	12/28	χ2(1) = 0.58,* p* = 0.45
Sex (females), *N* (%)	16 (40%)	16 (40%)	*χ*^*2*^(1) = 0.00, *p* = 1.00
IQ^a^	104.06 (11.19)	106.06 (11.25)	*t*(78) = -0.80, *p* = 0.43
IQ range	75–128	78–135	
Matrix reasoning	10.40 (2.35)	10.28 (2.71)	*U* = 761.00, *p* = 0.71
Vocabulatory test^b^	11.28 (2.95)	12.00 (3.32)	*t*(77) = -1.03, *p* = 0.31
Handedness, right/left, *N*	34/6	35/5	*χ*^*2*^(1) = 0.11, *p* = 0.75
Y(A)SR			
Internal	59.78 (10.63)	44.53 (11.04)	*U* = 277.5, *p* < 0.001
External	57.53 (8.15)	44.33 (8.43)	*t*(78) = 7.12, *p* < 0.001
Attention problems	66.83 (10.16)	53.40 (4.48)	*U* = 168.5, *p* < 0.001
ADHD rating scale^c^			
Inattention	15.38 (3.96)	n/a	
Hyperactive/impulsive	9.67 (5.03)	n/a	
Depressive symptoms			
IDS-C30	15.05 (12.63)	2.75 (3.97)	*U* = 184.0, *p* < 0.001, *R*^2^ = 0.44
Range	0–57	0–19	
BDI-II	11.53 (10.58)	2.15 (3.21)	*U* = 254.5, *p* < 0.001, *R*^2^ = 0.35
Range	0–42	0–12	

Mean values are shown. Standard deviations are given in parenthesis. The Mann–Whitney-*U* test was used in case data was not normally distributed in patients and/or healthy controls (HC). *ASR* Adult Self-Report [72], *BDI-II* Beck Depression Inventory II [74], *IDS-C30* Inventory of Depressive Symptomatology [73], *YSR* Youth Self-Report [71]

^a^Verbal and nonverbal intelligence were estimated by the vocabulary and matrix reasoning subtests of the Wechsler Adult Intelligence Scale [84] in adults and the Intelligence Scale for Children [85] in adolescents. Standard scores for each subtest and the mean IQ calculated across both tasks are reported

^b^This test was not conducted in one participant due to language problems

^c^DCL-ADHD from the DISYPS-II [118] for adolescents and ADHS-DC-Q from HASE [119] for adults

All patients met diagnostic criteria for ADHD (22 combined subtype, 18 predominantly inattentive subtype) according to the Diagnostic and Statistical Manual of Mental Disorders, Fifth Edition [1]. The diagnoses of ADHD and psychiatric comorbidities were established by performing structured clinical interviews by trained clinicians (K-SADS-PL for adolescents [69]; DIVA 2.0 for adults [70]). 19 patients (48%) suffered from at least one current co-morbid psychiatric condition (Table 2). More than one co-morbid condition (up to 4) were diagnosed in six patients (15%). Co-morbid conditions included affective disorders (current, *N* = 14 or lifetime, *N* = 4), anxiety disorders (*N* = 6), obsessive-compulsive disorder (*N* = 3), conduct disorder (*N* = 2), tic disorder (*N* = 1), and Borderline personality disorder (*N* = 1). 28 patients (70%) were currently treated with ADHD-relevant medication and eight patients received at least one antidepressant. HC had no psychiatric diagnosis, no family history of ADHD, and were medication-free [see Supplementary Information (SI1)]. The study was approved by the ethical commission of the Medical Faculty, Goethe University, Frankfurt, Germany. All participants gave written informed consent in accordance with the Declaration of Helsinki.

**Table 2:** Psychiatric comorbidities

Current co-morbid diagnosis	48%
Affective disorders (current or past)	45%
Major depressive disorder, single episode	3
Major depressive disorder, single episode, in full remission	2
Major depressive disorder, recurrent	9
Major depressive disorder, recurrent, in full remission	2
Persistent depressive disorder	2
Anxiety disorders	
Social anxiety disorder	4
Panic disorder	1
Agoraphobia	1
OCD	3
Persistent motor and vocal tic disorder	1
Conduct disorder	2
Borderline personality disorder	1

Number of patients and percentage of all patients (%) are given. For affective disorders, current and past diagnoses are listed; for all other disorders, current diagnoses are listed

*OCD* Obsessive-compulsive disorder

## Measures

### Severity of ADHD and depressive symptoms

Trained experts rated the severity of ADHD symptoms based on information from the clinical interviews (K-SADS-PL; DIVA 2.0, see SI2 for details). Participants also completed the attention problems subscale of the Youth Self-Report (YSR) and the Adult Self-Report (ASR) [71, 72]. The severity of current depressive symptoms was rated by a trained clinician using the Inventory of Depressive Symptomatology (IDS-C30) [73] and participants completed the Beck Depression Inventory (BDI-II) [74].

### Cognitive emotion regulation questionnaire

The CERQ [75] is a self-report questionnaire consisting of 36 items to measure the habitual use of 5 adaptive and 4 maladaptive cognitive emotion regulation strategies [75, 76] (see SI2). Individual subscales load on two factors, i.e., „adaptive strategies“ (mean sum score of the subscales *putting into perspective, positive refocusing, positive reappraisal, acceptance, and planning*) and „maladaptive strategies“ (mean sum score of the subscales *self-blame, other-blame, rumination, catastrophizing*) [75].

### Ambiguous cue-conditioning paradigm

#### Stimuli, task, and procedure

**Fig. 1:**
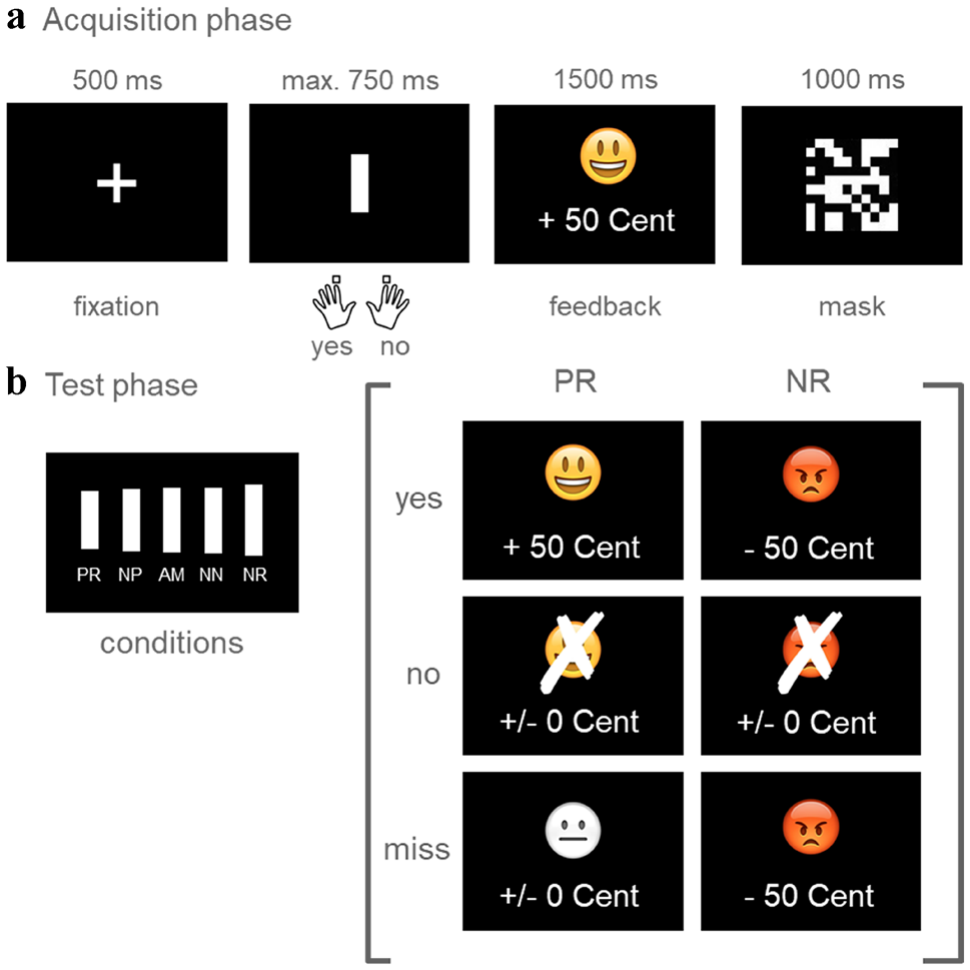
Ambiguous cue-conditioning paradigm. **a** Trial sequence in the acquisition phase, **b** Cue conditions in the test phase. Each trial started with a central fixation cross and the presentation of a reference bar (PR or NR) in the centre of the computer screen. Participants were instructed to understand each bar as an offer and to accept or reject the presented bar via pressing the “yes” or “no” button. Immediately afterwards, they received a feedback on the consequences of their responses followed by a central mask. When participants accepted the PR bar, they saw a smiley indicating a monetary gain (0.50 €), for rejection of the PR bar, they saw a crossed smiley indicating that they had missed the chance to earn money. The rejection of the NR bar was followed by a picture of a crossed frowney indicating that they had successfully avoided losing money. When they accepted the NR button, participants lost money (− 0.50 €) and saw a frowney. If participants did not press any button within the response window, they either lost money when the NR was presented or missed the chance to win money when the PR was presented. With this procedure, participants learned that the PR signaled the chance to win money when response “yes” was given, and the NR signaled the risk to lose money that could be avoided when response “no” was given. During the test phase, NP, AM, and NN bars were presented along with the bars from the acquisition phase (PR, NR). Participants were instructed to respond to each bar by pressing the “yes” (accepting) or “no” (rejecting) button. No feedback was given. Apart from the feedback, the presentation sequence of the test phase was identical to that of the acquisition phase. *PR* positive reference, *NP* near positive, *AM* ambiguous cue, *NN* near negative, *NR* negative reference

The ambiguous cue-conditioning paradigm is an indirect measure of cognitive bias based on evaluative conditioning. The paradigm has been developed in animal studies [77–79] and translated for human research [80–83]. In the current study, we implemented a visual version of the task (Fig. 1a, b) which was adapted to previous studies using auditory cues [82, 83]. Stimuli were presented and responses collected on a PC running the software Presentation (Neurobehavioral Systems). As shown in Fig. 1b, five bars of gradually increasing lengths were presented (viewing distance: 60 cm). The shortest (4.295° visual angle) and the longest (5.248°) bars served as reference stimuli, one as cue for a monetary gain (positive reference, PR) and one as cue for a monetary loss (negative reference, NR). The three intermediate bar lengths were ambiguous cues, labeled as near-positive (NP, 7% larger than the shortest bar; 4.6°), ambiguous (AM, 4.772°), and near-negative bar (NN, 7% smaller than the longest bar; 4.886°). The NP and the NN bars were partially ambiguous. They were visually more similar to either PR or NR. The AM bar was fully ambiguous; it was visually equidistant to PR and NR.

As shown in Fig. 1, the task included an acquisition and a testing stage. In the acquisition stage (Fig. 1a), participants learned to associate bars of different lengths (short vs. long) with positive and negative outcomes. Each trial started with a central fixation cross (500 ms) and the presentation of a reference bar (PR or NR) in the centre of the computer screen (maximum duration: 750 ms). Participants were instructed to understand each bar as an offer and to accept or reject the presented bar via pressing the “yes” or “no” button using their left or right index finger (counterbalanced across participants). Immediately afterwards, they received a feedback (1500 ms) on the consequences of their responses. When participants accepted the PR bar, they saw a smiley indicating a monetary gain (+ 0.50 €), for rejection of the PR bar, they saw a crossed smiley indicating that they had missed the chance to earn money. The rejection of the NR bar was followed by a picture of a crossed frowney indicating that they had successfully avoided losing money. When they accepted the NR button, participants lost money (− 0.50 €) and saw a frowney. If participants did not press any button within the response window, they either lost money when the NR was presented or missed the chance to win money when the PR was presented. With this procedure, participants learned that the PR signaled the chance to win money when response “yes” was given, and the NR signaled the risk to lose money that could be avoided when response “no” was given. The feedback was followed by a 1000-ms central mask (10.475° × 10.475°). The acquisition stage consisted of two blocks, each comprising 30 trials (15 trials for each reference length). If performance in the second block was lower than 90% correct, participants performed additional blocks of trials until 90% correct responses was reached within one block. Within each block, trial order was randomised for each individual with the constraint that each block contained the same number of trials for each bar length. The mapping of feedback to bar length and response to button was counter-balanced across participants with the constraint that each of the four possible combinations appeared equally often.

In the subsequent test phase, NP, AM, and NN bars were presented along with the bars from the acquisition phase (PR, NR) (Fig. 1b). No feedback was given in order to preclude that learning processes could influence the choice behaviour in the test phase. Participants were instructed to respond to each bar by pressing the “yes” (accepting) or “no” (rejecting) button. They were informed that trials would have no feedback and that they could win up to 12 € in one session. Apart from the feedback, the presentation sequence of the test phase was identical to that of the acquisition phase (Fig. 1a). The test phase comprised two sessions, each containing four blocks of 30 trials each (6 trials per condition), yielding a total of 240 trials (48 trials per condition). Within each block, trial order was randomised for each individual with the constraint that each block contained the same number of trials for each bar length. After each session, participants were informed about their winnings. They received the higher amount out of the two sessions.

### Statistical analyses

Group differences in measures of sample characteristics and clinical assessments were compared by t-tests (two-tailed) for continuous variables and by chi-square for categorical data. The Mann-Whitney-*U* test (two-tailed) was used in case the normality assumption was violated.

### CERQ

Group means in the CERQ maladaptive and adaptive total scores were compared with a multivariate analysis of variance (MANOVA) and subsequent univariate analyses of variance (ANOVAs). Group differences in each of the five adaptive and the four maladaptive subscales were explored with separate Mann-Whitney*U* tests (one-tailed) due to the directional nature of the hypotheses. Bonferroni correction was used to correct for multiple comparisons (threshold corrected for nine tests: *p* = 0.0055; see SI3).

In patients, we explored the relationship between the use of adaptive and maladaptive strategies and current ADHD and depression symptomatology. ADHD rating scores (total, inattentive subscale, and hyperactivity/impulsivity subscale scores), the attention problems subscale score of the YSR/ASR, as well as IDS-C30, and BDI-II total sores were analysed with separate linear regression models including adaptive and maladaptive CERQ total scores as predictors. All models were adjusted for ADHD medication (yes/no), other medication (yes/no), age, and IQ (estimated with vocabulary and matrix reasoning subtests [84, 85]). The assumption of normality of the distribution of residuals was fulfilled for all models (Shapiro-Wilk test, all *p*-values > 0.05).

### Ambiguous cue-conditioning paradigm

Bias scores were calculated as the mean of accept (coded as 1) and reject (coded as − 1) responses by condition (PR, NP, AM, NN, NR), resulting in bias scores ranging from -1 (perfectly negatively biased) and 1 (perfectly positively biased) with 0 representing no bias.

Separate two-way repeated-measures ANOVAs with a group factor (patients vs. HC) and the within-subject factor cue condition (NR, NN, AM, NP, PR) were conducted on bias scores and reaction times (RT; see SI3 and SI4). Our main hypothesis explicitly referred to a more negative interpretation of the ambiguous cue (AM) in patients vs. HC as indicated by a lower bias score for patients vs. HC in this condition. To preview, bias scores in the AM condition did not differ between groups. This null-result was additionally statistically evaluated by conducting Bayesian undirected independent samples Mann-Whitney-*U* test using JASP [86]. Bayes factors (BF_01_) were reported as the natural logarithm of the odds of the null hypothesis (H_0_) over the alternative hypothesis (H_1_). For Bayesian *t*-tests, we used the default prior on effect size (Cauchy distribution, centered on zero, with rate *r* = 0.707).

In patients, we explored the relationship between the individual bias score in the AM condition and ADHD and depression symptomatology with separate linear regression models (including clinician-based ratings and self-reports). All models were adjusted for ADHD medication (yes/no), other medication (yes/no), age, and IQ. The assumption of normality of the distribution of residuals was fulfilled for all models (Shapiro-Wilk test, all *p*-values > 0.14).

## Results

### Severity of depressive symptoms

The severity of depressive symptoms was significantly higher in patients vs. HC (Table 1). 19 patients scored above the clinical cut-offs of the BDI-II (> 9) and the IDS-C30 (> 11), in contrast to three HC with scores above the clinical cut-off of the BDI-II and two HC with scores above the clinical cut-off of the IDS-C30. Self-reported and clinician-rated depressive symptoms were strongly positively correlated both for patients (Spearman’s correlation coefficient: *r* = 0.64, *p* < 0.001) and HC (*r* = 0.60, *p* < 0.001). The severity of depressive symptoms was also significantly higher in patients vs. HC when patients with a depression diagnosis (*N* = 18) were excluded (IDS-C30, *M* = 10.41, *SD* = 9.63 for patients, *U* = 134.5, *p* < 0.001; BDI-II, *M* = 7.0, *SD* = 6.92 for patients, *U* = 199.0, *p* < 0.001).

### CERQ

Patients reported more frequent use of maladaptive strategies and less frequent use of adaptive strategies compared to HC (Table 3). A MANOVA on the total scores of the factors maladaptive and adaptive strategies yielded a significant main effect of group [*F*(2,77) = 11.47, *p* < 0.001, Wilks’ *λ* = 0.77, *ε*^2^ = 0.23]. Subsequent ANOVAs indicated a significant main effect of group for maladaptive strategies [*F*(1,78) = 17.48, *p* < 0.001, *ε*^2^ = 0.18]. A group effect was also found when patients with a depression diagnosis were excluded (see SI3-4). With regard to adaptive strategies, the group difference was marginally significant [*F*(1,78) = 3.97, *p* = 0.05, *ε*^2^ = 0.05]. There was no significant group difference in the use of adaptive strategies when patients with a depression diagnosis were excluded (see SI3-4).

**Table 3:** Use of adaptive and maladaptive cognitive emotion regulation strategies (CERQ)

	ADHD	HC	Test statistic
Maladaptive strategies^a^	10.08 (2.18)	8.07 (2.12)	*F*(1,78) = 17.48, *p* < 0.001, *ε*^2^ = 0.18
Adaptive strategies^a^	12.11 (2.79)	13.27 (2.38)	*F*(1,78) = 3.97, *p* = 0.05, *ε*^2^ = 0.05
Self-blame^b^	11.63 (3.23)	9.23 (3.25)	*U* = 471.0, *p* < 0.001, *R*^2^ = 0.13
Rumination	12.85 (3.96)	10.55 (4.41)	*U* = 518.0, *p* = 0.003, *R*^2^ = 0.09
Catastrophizing	8.18 (3.49)	6.05 (1.95)	*U* = 474.0, *p* = 0.001, *R*^2^ = 0.13
Blaming others	7.68 (3.04)	6.45 (2.10)	*U* = 603.0, *p* = 0.028, *R*^2^ = 0.05
Acceptance	13.40 (3.97)	13.35 (3.37)	*U* = 787.0, *p* = 0.45, *R*^2^ = 0.00
Positive refocusing	10.50 (4.01)	11.28 (3.47)	*U* = 697.5, *p* = 0.16, *R*^2^ = 0.01
Refocus on planning	12.20 (3.77)	13.45 (3.70)	*U* = 616.0, *p* = 0.038, *R*^2^ = 0.04
Positive reappraisal	12.33 (4.50)	14.65 (3.27)	*U* = 514.0, *p* = 0.003, *R*^2^ = 0.10
Putting into perspective	12.13 (3.55)	13.55 (3.71)	*U* = 610.5, *p* = 0.034, *R*^2^ = 0.04

Mean values are reported. Standard deviations are given in parenthesis. ^a^The MANOVA revealed a significant main effect of group [*F*(2,77) = 11.47, *p* < .001, Wilks’ λ = 0.77, *ε*^2^ = 0.23], which was followed up by ANOVAS. ^b^Mann-Whitney-*U* tests (one-tailed) were used to explore group differences on individual subscales (Bonferroni corrected threshold for nine tests: *p* = 0.0055). *HC* healthy controls

With regard to individual subscales, patients reported significantly more frequent use of self-blame (*U* = 471, *p* < 0.001, one-tailed), catastrophizing (*U* = 474, *p* = 0.001, one-tailed), and rumination (*U* = 518, *p* = 0.003, one-tailed). They also reported less frequent use of positive reappraisal (*U* = 514, *p* = 0.003, one-tailed). Patients did not significantly differ on any of the other subscales (Table 3). When patients with a depression diagnosis were excluded, we found no significant group differences in individual maladaptive subscales (see SI3-4).

### Association between emotion regulation strategies and ADHD symptomatology in patients

None of the linear regression models yielded a significant association between either CERQ total scores for adaptive or maladaptive strategies and severity of ADHD symptomatology (model fits: all *R*^2^-values < 0.24, all *F*-values < 1.8, all *p*-values > 0.13, Supplementary Table 1).

### Association between emotion regulation strategies and depressive symptomatology in patients

Linear regression models indicated that higher scores for CERQ maladaptive strategies (*β* = 0.35, *p* = 0.025) and lower scores for CERQ adaptive strategies (*β* = − 0.27, *p* = 0.032) were associated with higher scores on the IDS-C30 rating scale (model fit: *R*^*2*^ = 0.54, *F*(6,39) = 6.45, *p* < 0.001). Lower CERQ total scores for adaptive strategies were also associated with higher BDI-II total scores (*β* = − 0.33, *p* = 0.015; model fit: *R*^2^ = 0.48, *F*(6,39) = 5.07, *p* = 0.001). The total score for CERQ maladaptive strategies did not predict BDI-II total score (*β* = 0.139, *p* = 0.38, Supplementary Table 2).

### Ambiguous cue-conditioning paradigm

Participants were able to discriminate the two reference cues (NN, PR) as indicated by 94.67% (*SD* = 3.10) and 93.83% (*SD* = 3.16) correct responses in the last training session for patients and HC, respectively (*U* = 677.00, *p* = 0.21). Patients and HC did not differ in the mean number of training sessions [2.95 (*SD* = 1.13, range 2–8) for patients; 3.33 (*SD* = 1.70, range 2–9) for HC; Mann-Whitney *U* = 742.00, *p* = 0.56]. Although performance dropped in the test phase when presenting additional intermediate cues [86.63% (*SD* = 10.31) for patients, 90.67% (*SD* = 7.02) for HC; significant difference between training and test session: Wilcoxon rank sum test, *Z* = − 4.07, *p* < 0.001 for patients; *Z* = − 2.31, *p* < 0.05 for HC], the percentage of correct responses to the reference cues was still high and did not significantly differ between groups (*U* = 619.00, *p* = 0.08).

**Fig. 2:**
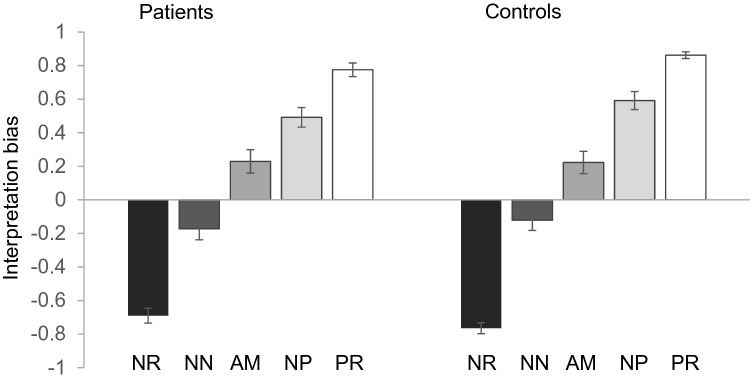
Results in the ambiguous cue-conditioning paradigm: Mean interpretation bias score as a function of cue condition. *PR* positive reference, *NP* near positive, *AM* ambiguous cue, *NN* near negative, *NR* negative reference

Figure 2 displays the bias scores for all cue conditions separately for patients and HC (see SI4 and Supplementary Fig. 1 for RT). A two-way repeated-measures ANOVA revealed a significant main effect of cue condition [*F*(2.76,215.23) = 525.26, *p* < 0.001, *ε*^2^ = 0.87] on bias scores. The main effect of group [*F*(1,78) = 0.29, *p* = 0.599, *ε*^2^ = 0.004] and the group *x* condition interaction [*F*(2.76, 215.23) = 1.872, *p* = 0.14, *ε*^2^ = 0.023] were not significant. Similarly, the robust repeated-measures ANOVA yielded a significant main effect of cue condition (*p* < 0.001) but no effect of group (*p* = 0.52) and no interaction effect between the two factors (*p* = 0.26). As indicated by pairwise follow-up comparisons calculated across both groups, bias scores significantly differed between NR and NN (*Z* = − 7.77, *p* < 0.001), NN and AM (*Z* = − 7.60, *p* < 0.001), AM and NP (*Z* = − 7.42, *p* < 0.001), and NP and PR (*Z* = − 7.17, *p* < 0.001). As indicated by the ANOVAs, bias scores in the AM condition did not differ between groups (0.229, *SD* = 0.44 for patients; 0.223, *SD* = 0.42 for HC) (Supplementary Fig. 2). When conducting a Bayesian independent samples Mann-Whitney-*U* test, calculation of the Bayes factor (BF_01_) yielded 4.26 times stronger support for the null hypothesis of no group difference in the bias score in the AM condition over the alternative hypothesis.

### Association between interpretation bias and ADHD symptomatology in patients

None of the linear regression models yielded a significant association between the bias score in the AM condition and severity of ADHD symptomatology (model fits: all *R*^2^-values < 0.24, all *F*-values < 2.1, all *p*-values > 0.09, Supplementary Table 3).

### Association between interpretation bias and depressive symptomatology in patients

Linear regression models revealed no significant association between bias scores in the AM condition and total scores in self-rated (BDI-II, *β* = − 0.06, *t* = − 0.42, *p* = 0.68) and clinician-rated (IDS-C30, *β* = − 0.10, *t* = − 0.68 *p* = 0.50) depressive symptomatology (Supplementary Table 4).

## Discussion

Our findings provide new insights into impairments in cognitive emotion regulation in youth with ADHD and their role in the development of co-morbid depression. With respect to the explicit measure of cognitive emotion regulation, youth with ADHD who are at increased risk for co-morbid depression reported more frequent use of maladaptive and less frequent use of adaptive cognitive emotion regulation strategies. This finding is consistent with the depression literature [40, 87]. Furthermore, the pattern of strategy use in daily life predicted the severity of current depressive but not ADHD symptoms in patients. On the other hand, for the implicit measure of cognitive bias, we found no evidence for a bias towards negative interpretations of ambiguous information in youth with ADHD. This finding is in conflict with the literature associating depression [36, 49] and depression risk [33–35] with a cognitive bias favouring negative interpretations. The individual interpretation bias as assessed with the ambiguous-cue conditioning task was neither significantly associated with current depressive nor with ADHD symptoms. Together, these findings point to depression-related alterations in the use of cognitive emotion regulation strategies in youth with ADHD when measured with self-reports that require explicit awareness into regulatory processes.

The more frequent use of CERQ maladaptive strategies which we observed in youth with ADHD - even when patients with a depression diagnosis were excluded - is partly consistent with similar reports in adults with ADHD [57]. In the present sample, the effect was mostly driven by the strategies self-blame, catastrophizing, and rumination. Self-blame and catastrophizing are two strategies that have been related to self-reported depressive symptoms in clinical and general population samples [42, 75, 76, 88, 89] and may distinguish best between patients with depression and HC [42, 90]. In addition, rumination, has been consistently associated not only with depressive symptoms [75, 76, 88, 89] but also depression risk [38, 40, 45]. Recent evidence from a cross-sectional study suggests that the tendency to ruminate may predict the development of co-morbid depression in adults with ADHD [58]. The present findings add to this evidence by suggesting that a similar association may exist in youth with ADHD. With regard to blaming others, the group difference was not significant - a finding that is also consistent with depression research pointing to no association between the use of this strategy and depressive symptomatology [75, 76, 89] or depression diagnosis [42, 90]. With regard to adaptive strategies, we found a significant group difference for positive reappraisal when all patients were included in the analysis but not when excluding patients with a depression diagnosis. Similar to patients with depression [40, 87] as well as adolescents and adults from the general population with high levels of depressiveness [75, 76, 88, 89], youth with ADHD may use positive reappraisal less often than HC. However, it remains to be determined to what degree alterations in their use of adaptive strategies [57, 91] are a manifestation of current depression comorbidity and/or depression risk. Together, these findings point to a pattern of depression-related cognitive emotion regulation strategies (i.e., including high levels of self-blame, catastrophizing, and rumination, and low levels of positive reappraisal) in youth with ADHD, which may explain - at least partly - their increased risk to develop co-morbid depression during the critical developmental window of adolescence and early adulthood.

Why would the occurrence of ADHD provoke a risk pattern of cognitive emotion regulation making patients more vulnerable for depression? Given that executive functions strongly interact with emotional processing in the aetiology of depression and depression risk [92, 93], impairments in executive functions, which are a central characteristic of ADHD [94] and depression [95], may contribute to differences in the use of cognitive emotion regulation strategies and depression risk in patients with ADHD. The effective use of reappraisal has been associated with better performance in tasks on working memory [96, 97] and verbal fluency [98]—two markers of executive functions that are candidate neurocognitive risk markers of ADHD-depression comorbidity [95]. In addition, the extent to which positive reappraisal is used increases from adolescence to adulthood [76], probably reflecting a refinement of cognitive strategies of emotion regulation that depends on maturation of executive functions and the development of prefrontal cortex [99, 100]. The maturation of the prefrontal cortex continues into adolescence [101] and young adulthood [102] but is delayed in adolescents with ADHD [103]. This may hinder the development of complex strategies and increase vulnerability to depression during the highly sensitive phase of adolescence/early adulthood in patients with ADHD. While deficits in executive functions may render the use of adaptive strategies more difficult, they could also increase the use of maladaptive strategies. In line with this hypothesis, rumination has been linked to impaired cognitive inhibition in patients with depression [104, 105]. Besides these executive dysfunctions, long-term memory deficits - another marker of structural brain abnormalities - have been associated with poor emotion regulation in adolescents with depression [106]. Given that long-term memory deficits are also part of the overlapping neurocognitive profiles of ADHD and depression [95], these deficits may also influence cognitive emotion regulation and increase depression risk in youth with ADHD.

While the effective use of cognitive emotion regulation strategies was significantly associated with lower levels of current depressive symptoms in patients, we found no association with the severity of ADHD symptoms, both when assessed with self-reports and clinician-based ratings. These findings suggest that the identified alterations in the use of adaptive and maladaptive strategies were linked to depressive rather than core ADHD symptoms. This finding does not exclude that other aspects of emotion dysregulation (e.g., increased emotional reactivity/impulsivity [24, 25, 107] may reflect a fundamental aspect of ADHD per se [29, 108, 109].

The implicit measure of cognitive bias implemented in this study has originally been developed in the context of animal models of depression [77–79] and translated for human research [80–83]. Previous studies in humans have consistently reported an association between a more negative interpretation bias and elevated levels of depressiveness as well as experimentally induced depressive mood in general population samples [80–83]. Four our clinical sample of youth with ADHD, however, we did not find evidence for a more negative interpretation bias. The individual scores of biased information processing were also not significantly associated with the severity of current depressive symptoms. These findings do not suggest that depression-related alterations in interpretation of ambiguous information can be considered as part of the cognitive risk profile of depression co-morbidity among youth with ADHD as compared to HC. However, these findings need to be interpreted with caution. Albeit carefully matched for demographical variables, the present sample size may have provided insufficient power for detecting small effects. With *N* = 40 participants per group, the study allowed us to detect a moderate to large group effect (Cohen’s *d* = 0.63) at an *α*-level of *p* < 0.05 with a power of 0.8 in a two-sided *t*-test [110]. Furthermore, the Bayes factor calculated for the group comparison in the ambiguous cue-conditioning paradigm (BF_01_ = 4.26) provided only moderate evidence for the null hypothesis of no group difference in interpretation bias. Thus, while our study does not suggest a difference in the interpretation of ambiguous information by youth with ADHD compared to HC, our findings do not rule out smaller effects that might be detected by higher-powered studies.

The following limitations need to be considered for the current study. Due to the cross-sectional nature of the study, it is not clear whether the identified differences in cognitive emotion regulation causally contribute to the development of depression. To answer this question longitudinal studies are needed that examine if alterations in cognitive emotion regulation strategies occur before a depressive episode and prospectively predict its onset in youth with ADHD. Also, we cannot exclude that group findings were influenced by sample characteristics. The patient sample was comparable to previous reports in terms of depression rate and the majority of patients was medicated. Taking medication into account by adjusting regression models for ADHD and other medication, the frequencies of adaptive and maladaptive strategies were associated with depressive symptoms in patients. However, in the light of potentially protective effects of ADHD medication against the occurrence and development of depression [111], and positive effects of stimulants [63] and antidepressive medication on emotion regulation [112], group differences in cognitive emotion regulation may have been underestimated in this medicated sample. Therefore, group findings may generalise largely to medicated samples. Finally, the abstract geometric figures used as stimuli in the ambiguous cue-conditioning paradigm and the monetary incentives used as reinforcers may have had low relevance to the participants. Future studies could aim at implementing task variants with higher ecological validity by including self-referential information or social reinforcers, which are of particular importance to patients with depression [36, 113]. This may refine our understanding of biases in the interpretation of ambiguous information in ADHD and depression.

In conclusion, the present findings suggest that youth with ADHD have difficulties in the effective use of cognitive emotion regulation strategies, which are linked to their current depressive symptomatology. These deficits in cognitive emotion regulation may also confer a risk to develop later depression among patients with ADHD during the critical developmental window of adolescence and early adulthood. These findings highlight an important candidate risk factor for ADHD-depression comorbidity which should be integrated in future longitudinal studies on comorbidity in ADHD.

For the clinical practice, these findings point to the importance of screening for the occurrence of co-morbid depression in adolescents and young adults with ADHD but also monitoring their risk to develop depression. Specifically, the assessment of cognitive emotion regulation strategies in young patients may help therapists to better detect co-morbid depression that often presents in a ruminative quality among youth with ADHD [114]. Furthermore, assessing the use of cognitive emotion regulation strategies in daily life may help clinicians to identify individuals who are at highest risk for future depression. This would provide important opportunities for early intervention and prevention strategies that are not available at present. Our findings suggest that improving cognitive emotion regulation strategies should be considered as an important target of treatment of ADHD-depression comorbidity, for example by adapting cognitive strategies that are core components of Cognitive Behaviour Therapy in adults [115], to programmes for youth with ADHD and depression [116, 117]. Furthermore, modification of cognitive emotion regulation strategies before clinical manifestation of depression could be beneficial for youth with ADHD to protect them against onset of depression in the first place and thus prevent a more adverse course of ADHD.

## Supplementary Information

Below is the link to the electronic supplementary material.

Supplementary file1 (DOCX 139 KB)

## Data Availability

Data are available from the correspondence author.
