# Supplementary material for: Depressive symptoms in youth with ADHD: the role of impairments in cognitive emotion regulation
Source: Eur Arch Psychiatry Clin Neurosci. 2022 Feb 2;272(5):793–806. doi: 10.1007/s00406-022-01382-z (PMC9279209; doi:10.1007/s00406-022-01382-z)
Supplement: Supplementary file 1 — Supplementary file1 (DOCX 139 KB) [file 406_2022_1382_MOESM1_ESM.docx]

**Supplementary Information**

**Title: Depressive symptoms in youth with ADHD: the role of impairments in cognitive emotion regulation**

**Journal:** European Archives of Psychiatry and Clinical Neuroscience

**Authors:** Mayer, J.S., Brandt, G.A., Medda, J., Basten, U., Grimm, O., Reif, A., Freitag, C.M.

**Corresponding author:**

Dr. Jutta S. Mayer

Department of Child and Adolescent Psychiatry, Psychosomatics and Psychotherapy, University Hospital Frankfurt, Goethe University, Deutschordenstraße 50, 60528 Frankfurt am Main, Germany, email: [jutta.mayer@kgu.de](mailto:jutta.mayer@kgu.de)

**SI1: Diagnostic procedures and exclusion criteria**

The Kiddie-Schedule for Affective Disorders and Schizophrenia - Present and Lifetime Version (K-SADS-PL) [1] was used to assess ADHD symptoms and co-morbid psychiatric conditions in adolescents. The K-SADS-PL is a semi-structured diagnostic interview designed to assess current and past episodes of psychopathology in children and adolescents according to DSM-IV criteria [1]. Diagnosis of ADHD was adjusted to DSM-5 criteria. To obtain as much information as possible for high valid diagnoses, both with regard to externalizing and internalizing conditions, the K-SADS-PL was conducted separately with the adolescent and one primary caregiver. For the final rating, the trained clinician took both sources of information into account. The German version of the K-SADS-PL shows good validity in ADHD diagnoses [2]. To assess ADHD symptoms in adults, the Diagnostic Interview for ADHD in adults (DIVA 2.0) [3] was administered by a trained clinician. The DIVA is a structured diagnostic interview to investigate the DSM-IV criteria of ADHD in childhood and adulthood, as well as impairment in five areas of functioning in both life periods. In order to simplify the evaluation of each of the 18 symptom criteria for ADHD, in childhood and adulthood, the interview provides a list of concrete and realistic examples, for both current and retrospective (childhood) behaviour. Diagnosis of ADHD was adjusted to DSM-5 criteria. The DIVA shows good diagnostic validity in adults [4, 5]. Furthermore, adult patients filled out the Adult ADHD Self-Report Scale Symptom Checklist (ASRS) [6]. This is a self-report symptom checklist developed by the World Health Organization that includes 18 questions of recent DSM-IV Criterion A symptoms of adult ADHD. Participants are asked how often a symptom has occurred over the past 6 months on a scale ranging from 0 to 4 (never, rarely, sometimes, often, very often). 26 adult patients (84%) scored above the screening cut-off (≥ 4 for inattention). The German language versions of the Structured Clinical Interview for DSM-IV Axis I Disorders (SCID-I) and Axis II Disorders (SCID-II, only part on Borderline personality disorder) [7] were carried out with all adult patients by a trained clinical psychologist to assess psychiatric comorbidities. Diagnoses were adjusted to DSM-5 criteria.

Healthy controls (HC) were screened for current psychopathology using the Child Behavior Checklist (parent reports) [8] and the Youth Self-Report in adolescents (YSR) [9] and the Adult Self-Report (ASR) [10] in adults. No adolescent scored above clinical cut-off on any of the subscales. One adult scored above clinical cut-off on the internalizing and the social withdrawal subscales of the ASR, but the existence of any current DSM-5 Axis I disorder was ruled out using the SCID-I interview. Furthermore, adult participants were screened for ADHD using the ASRS. No control participant scored above the screening cut-off (≥ 4 for inattention). In addition, family history of ADHD was assessed using a semi-structured interview. HC with a family history of ADHD were excluded. Participants in the ADHD group who suffered from bipolar disorder, schizophrenia, schizoaffective or organic psychiatric disorder (current or lifetime) were excluded from participation. All participants had normal or corrected-to-normal vision. Common exclusion criteria for both groups were any severe medical or neurological condition (e.g. epilepsy), pregnancy, current substance abuse/ dependence, and an intelligence quotient below 75. Verbal and nonverbal intelligence were estimated by the vocabulary and matrix reasoning subtests of the Wechsler Adult Intelligence Scale [11] in adults and the Intelligence Scale for Children [12] in adolescents.

**Medication**

28 patients (70%) were currently treated with ADHD-relevant medication (Methylphenidate, *N* = 21; Lisdexamphetamine, *N* = 4; Dexamphetamine, *N* = 1; Atomoxetine, *N* = 1) and eight patients received at least one antidepressant (Fluoxetine, *N* = 2; Sertraline, *N* = 1; Venlafaxine, *N* = 1; Buproprione, *N* = 3; Mirtazapine, *N* = 1; Opipramole, *N* = 1, Trimipramine, *N* = 1, Trazodone, *N* = 1). Other medication included Tiapride (*N* = 1) to treat tic disorders, and Promethazine to treat sleep problems *(N* = 1). All medication was taken on a stable dosage for at least 4 weeks.

**SI2: Measures**

**Severity of ADHD symptoms**

The severity of ADHD symptoms was rated by trained experts based on information from the clinical interviews (K-SADS-PL for adolescents; DIVA for adults). For adolescent patients, we used the Diagnostic Checklist for Attention Deficit Hyperactivity Disorder (DCL-ADHD) from the DISYPS-II (Diagnostik-System für psychische Störungen nach ICD-10 und DMS-IV für Kinder und Jugendliche) [13]. For adult patients, we used the ADHD Diagnostic Checklist-Quantitative (ADHS-DC-Q) from the Homburger ADHD Scales for Adults [14]. Both scales are exactly comparable 18-item scales assessing ADHD symptoms by a 4-point Likert-type severity scale. Validity of these instruments as well as good inter-rater reliability (intra-class coefficient of 0.93 for ADHS-DC-Q) and internal consistency (Cronbach’s 0.96 for ADHS-DC-Q) have been reported [14].

Severity of ADHD symptoms was also assessed with the attention problems subscale of the YSR and the ASR, which are designed to be self-administered by youths aged 11-18 years and adults aged 18-59 years, respectively [9, 10]. The instruments allow an age and gender adjusted subjective measurement of psychopathology including 10 subscales comprising internalising and externalising behaviours. All items are rated on a 3-point Likert-type severity scale. For statistical use, the scores of the internalising and externalising behaviour scales were transformed into T-scores. Summary scores (internalising and externalising problems) as well as individual subscale scores were calculated. The attention problems subscale includes nine items. Acceptable internal consistencies (Cronbach’s α of 0.65-0.89 for subscales of YSR11-18R and 0.51-0.88 for subscales of ASR) and good validity have been shown in clinical and non-clinical samples [10, 15].

**Severity of depressive symptoms**

The severity of depressive symptoms was assessed in patients and HC. The severity of depressive symptoms was rated by a trained clinician using the Inventory of Depressive Symptomatology (IDS-C30) [16]. The IDS-C30 rating includes all DSM-5 diagnostic criterion items for major depressive disorder (e.g. mood, vegetative, psychomotor, and cognitive symptoms) as well as commonly associated symptoms such as anxiety, irritability, melancholic, and atypical symptom features to assess the severity of depressive symptoms over the last seven days. Items are rated on a 4-point Likert scale based on the information obtained during a semi-structured interview. The total score range is 0–84. Internal consistency of the German version has shown highly acceptable internal consistency (Cronbach’s α of 0.93) and good validity [17]. Furthermore, participants completed the Beck Depression Inventory (BDI-II) [18], which is a widely used self-report scale for individuals aged 13 years and older. It contains 21 items that are rated on a Likert (0-3 coded) scale. The German BDI-II demonstrates good validity and reliability in clinical (Cronbach’s α of 0.84) and nonclinical samples (Cronbach’s α of 0.89) [19].

**Cognitive emotion regulation questionnaire (CERQ)**

The nine CERQ subscales characterise the individual‘ s style of responding to negative events and include self-blame, other-blame, rumination, catastrophizing, putting into perspective, positive refocusing, positive reappraisal, acceptance, and planning. Each scale consists of four items measured on a 5-point Likert scale ranging from 1 (almost never) to 5 (almost always). Individual subscale scores are obtained by summing up the scores belonging to the particular subscale (ranging from 4 to 20). Internal consistencies for the subscales have been reported to range from Cronbach’s α = 0.68 to Cronbach’s α = 0.86 [20–22]. The German version of the CERQ has been validated and also shows acceptable internal consistency (Cronbach’s α = 0.60 to 0.86) [23].

**SI3: Statistical analyses**

**CERQ – Main analysis**

MANOVA assumptions (i.e., multivariate normality, homogeneity of error variances and of covariances) were fulfilled. Multivariate normality was tested with the Shapiro-Wilk test (*p* >.074). Homogeneity of error variances across groups was assessed using Levene’s test (all *p*-values > .31) and homogeneity of covariances were assessed by Box’s test (*p* = .65). To statistically compare group differences in each of the nine subscales, we used separate Mann-Whitney-U tests (one-tailed) because assumptions of multivariate normality (Shapiro-Wilk test, *p* < .001), homogeneity of covariances (Box’s test, *p* < .001), and homogeneity of error variances across groups (Levene’s test, *p* < .05 for catastrophizing and positive reappraisal, all other p-values > .05) were violated. Bonferroni correction was used to correct for multiple comparisons (threshold corrected for nine tests: *p* = .0055).

**CERQ – Subgroup analyses comparing ADHD patients without a comorbid depression diagnosis vs. HC**

Group means in the CERQ maladaptive and adaptive total scores were compared with a multivariate analysis of variance (MANOVA) and subsequent univariate analyses of variance (ANOVAs). Multivariate normality was not fulfilled (Shapiro-Wilk test, *p* < .05). Homogeneity of error variances across groups (Levene’s test, all *p*-values > .24) and homogeneity of covariances were given (Box’s test, *p* = .71). To statistically compare group differences in each of the four maladaptive subscales, we used Mann-Whitney-U tests (one-tailed) because the assumption of normality was violated in some conditions (Shapiro-Wilk test, *p* < .05). Bonferroni correction was used to correct for multiple comparisons (threshold corrected for four tests: *p* = .0125).

**Ambiguous cue-conditioning paradigm**

For each group and each factor level, dependent variables (bias sores and RTs) were assessed for normality using the Shapiro-Wilk-Test. Homogeneity of error variances across groups was assessed using Levene’s test and homogeneity of covariances were assessed by Box’s test. Greenhouse– Geisser corrected *p*-values were reported in cases where ANOVA sphericity assumptions were violated, which was determined with a Mauchly’s test for sphericity (*p* < 0.05). Because assumptions of normality distribution (Shapiro-Wilk-Test, *p*-values < .05), homogeneity of covariances (Box’s test, *p* = .003), homogeneity of error variances across groups (Levene’s test, *p* < .05 for NR and PR) were violated with regard to bias scores, we also conducted a robust two-way repeated measures ANOVA on bias scores using the WRS2 package [24] in R [25]. Significant main effects were followed up with Wilcoxon rank sum tests (for bias scores) or *t*-tests (RT).

**SI4: Results**

**CERQ – Subgroup analyses comparing ADHD patients without a comorbid depression diagnosis vs. HC**

ADHD patients without a depression diagnosis (*N* = 22) reported more frequent use of maladaptive emotion regulation strategies (*M* = 9.20, *SD* = 1.98) and less frequent use of adaptive strategies (*M* = 12.27, *SD* = 2.84) compared to HC (*M* = 8.07, *SD* = 2.12 for maladaptive, *M* = 13.27, *SD* = 2.38 for adaptive strategies). A MANOVA on the total scores of the factors maladaptive and adaptive emotion regulation strategies yielded a significant main effect of group [*F*(2,59) = 3.59, *p* < .05, Wilks’ λ = 0.89, ε^2^ = 0.11]. Subsequent ANOVAs indicated a significant main effect of group for maladaptive strategies [*F*(1,60) = 4.25, *p* < .05, *ε^2^* = 0.07]. With regard to adaptive strategies, the group difference was not significant [*F*(1,60) = 2.15, *p* = .15, *ε^2^* = 0.04].

With regard to individual maladaptive subscales, group differences were not significant when taken multiple comparisons into account (Bonferroni corrected threshold for four tests: *p* = .0125): blaming others (*U* = 295.5, *p* = .016, one-tailed), self-blame (*U* = 331, *p* = .054, one-tailed), catastrophizing (*U* = 330.5, *p* = .051, one-tailed), rumination (*U* = 375.5, *p* = .171, one-tailed).

**Ambiguous cue-conditioning paradigm: RT**

Supplementary Fig. 1 displays the RT data for all cue conditions separately for patients and HC. A two-way repeated-measures ANOVA revealed a significant main effect of cue condition [*F*(2.74, 235.34) = 41.40, *p* < .001, ε^2^ = 0.35], which points to RT differences between NR and NN [*t*(79) = -5.78, *p* < .001], AM and NP [*t*(79) = 3.87, *p* < .001], and NP and PR [*t*(79) = 8.99, *p* < .001] as indicated by pairwise follow-up comparisons. The difference between conditions NN and AM did not reach significance [*t*(79) = 2.05, *p* = .043, Bonferroni corrected threshold for four tests: *p* = .0125]. The main effect of group [*F*(1,78) = 0.114, *p* = .736, ε^2^ = 0.001] and the group x cue condition interaction [*F*(2.74, 213.34) = 0.652, *p* = .58, ε^2^ = 0.008] were not significant.


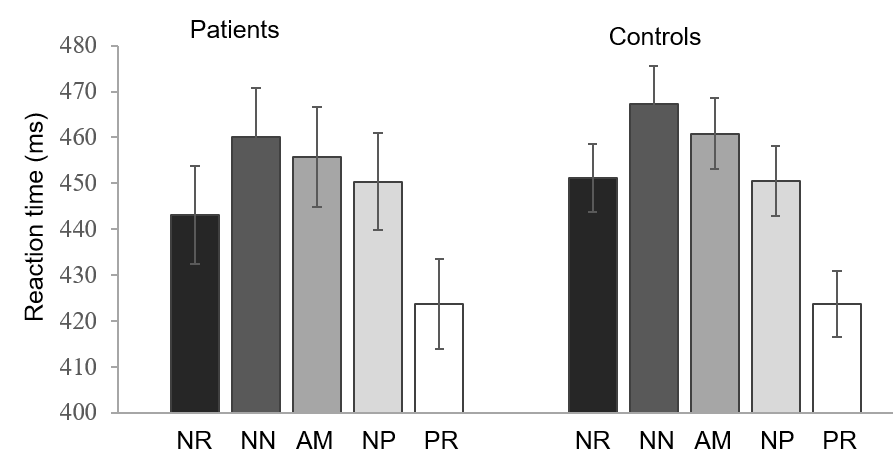


**Supplementary Fig. 1** Ambiguous cue-conditioning paradigm: Mean RT as a function of cue condition

PR = positive reference, NP = near positive, AM = ambiguous cue, NN = near negative, NR = negative reference

**Ambiguous cue-conditioning paradigm: Bias scores**

**
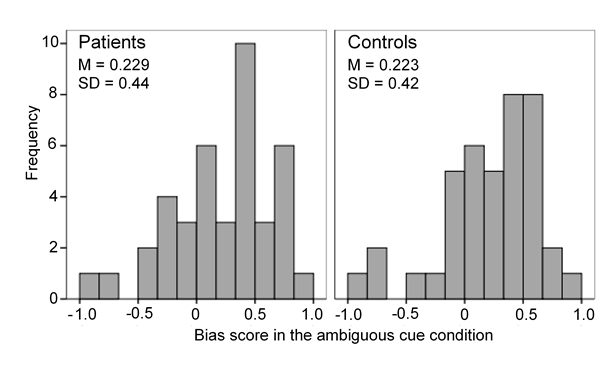
**

**Supplementary Fig. 2** Distribution of bias scores in the ambiguous-cue condition for patients and healthy controls

Mean values (*M*) and standard deviations (*SD*) are reported.

**Supplementary Table 1** Association between the use of adaptive and maladaptive emotion regulation strategies and severity of ADHD symptoms in patients

| Model criterion | Model summary | | ANOVA | |
| --- | --- | --- | --- | --- |
|  | *R^2^* | *Total R^2^ Adj* | *F* | *p* |
| ADHD rating scale total score | 0.095 | -0.075 | 0.561 | .758 |
| ADHD rating scale  inattentive subscale | 0.114 | -0.052 | 0.685 | .663 |
| ADHD rating scale  hyperactivity/ impulsivity subscale | 0.168 | 0.12 | 1.076 | .397 |
| YSR/ASR  attention problems subscale | 0.244 | 0.106 | 1.770 | .136 |

Results of linear regression models predicting ADHD symptoms are reported. All linear regression models included adaptive and maladaptive CERQ total scores as predictors and were adjusted for ADHD medication (yes/ no), other medication (yes/ no), age, and IQ.

**Supplementary Table 2** Association between the use of adaptive and maladaptive emotion regulation strategies and severity of depressive symptoms in patients

| Model criterion | Model summary | | ANOVA | | Predictor | Coefficients | | |
| --- | --- | --- | --- | --- | --- | --- | --- | --- |
|  | *R^2^* | *Total R^2^ Adj* | *F* | *p* |  | *β* | *t* | *p* |
| IDS-C30 total score | 0.540 | 0.456 | 6.453 | .000 | CERQ adaptive | -0.274 | -2.241 | .032 |
|  |  |  |  |  | CERQ maladaptive | 0.348 | 2.354 | .025 |
|  |  |  |  |  | Age | 0.316 | 2.207 | .034 |
|  |  |  |  |  | IQ | -0.252 | -1.911 | .065 |
|  |  |  |  |  | ADHD medication | 0.014 | 0.114 | .910 |
|  |  |  |  |  | Other medication | -0.012 | -0.088 | .930 |
| BDI-II total score | 0.480 | 0.385 | 5.070 | .001 | CERQ adaptive | -0.334 | -2.566 | .015 |
|  |  |  |  |  | CERQ maladaptive | 0.139 | 0.886 | .382 |
|  |  |  |  |  | Age | 0.315 | 2.058 | .047 |
|  |  |  |  |  | IQ | -.233 | -1.659 | .107 |
|  |  |  |  |  | ADHD medication | 0.061 | 0.472 | .640 |
|  |  |  |  |  | Other medication | .186 | 1.336 | .191 |

Results of linear regression models predicting ADHD symptoms are reported. All linear regression models included adaptive and maladaptive CERQ total scores as predictors and were adjusted for ADHD medication (yes/ no), other medication (yes/ no), age, and IQ.

**Supplementary Table 3** Association between bias score and severity of ADHD symptoms in patients

| Model criterion | Model summary | | ANOVA | |
| --- | --- | --- | --- | --- |
|  | *R^2^* | *Total R^2^ Adj* | *F* | *p* |
| ADHD rating scale total score | 0.098 | -0.039 | 0.714 | .617 |
| ADHD rating scale  inattentive subscale | 0.094 | -0.043 | 0.685 | .638 |
| ADHD rating scale  hyperactivity/ impulsivity subscale | 0.091 | -0.046 | 0.663 | .654 |
| YSR/ASR  attention problems subscale | 0.236 | 0.124 | 2.103 | .089 |

Results of linear regression models predicting ADHD symptoms are reported. All linear regression models included the bias score in the ambiguous cue condition as predictor and were adjusted for ADHD medication (yes/ no), other medication (yes/ no), age, and IQ.

**Supplementary Table 4** Association between bias score and severity of depressive symptoms in patients

| Model criterion | Model summary | | ANOVA | | Predictor | Coefficients | | |
| --- | --- | --- | --- | --- | --- | --- | --- | --- |
|  | *R^2^* | Total *R^2^* Adj | *F* | *p* |  | *β* | *t* | *p* |
| IDS-C30 total score | 0.397 | 0.308 | 4.471 | .003 | Bias score | -0.096 | -0.678 | .502 |
|  |  |  |  |  | Age | 0.388 | 2.527 | .016 |
|  |  |  |  |  | IQ | -0.294 | -1.994 | .054 |
|  |  |  |  |  | ADHD medication | 0.061 | 0.436 | .666 |
|  |  |  |  |  | Other medication | 0.150 | 1.057 | .298 |
| BDI-II total score | 0.365 | 0.271 | 3.904 | .007 | Bias score | -0.061 | -0.419 | .678 |
|  |  |  |  |  | Age | 0.316 | 2.005 | .053 |
|  |  |  |  |  | IQ | -.220 | -1.450 | .156 |
|  |  |  |  |  | ADHD medication | 0.071 | 0.500 | .621 |
|  |  |  |  |  | Other medication | .298 | 2.048 | .048 |

Results of linear regression models predicting depressive symptoms are reported. All linear regression models included the bias score in the ambiguous cue condition as predictor and were adjusted for ADHD medication (yes/ no), other medication (yes/ no), age, and IQ.

References

1. Kaufman J, Birmaher B, Brent D, Rao U, Flynn C, Moreci P, Williamson D, Ryan N (1997) Schedule for Affective Disorders and Schizophrenia for School-Age Children-Present and Lifetime Version (K-SADS-PL): initial reliability and validity data. Journal of the American Academy of Child and Adolescent Psychiatry 36(7):980–988. https://doi.org/10.1097/00004583-199707000-00021

2. Schmidt S, Banaschewski T, Garbe E, Petermann F, Petermann U (2013) Diagnostik der ADHS im Kindes- und Jugendalter mit dem K-SADS-PL. Praxis der Kinderpsychologie und Kinderpsychiatrie 62(7):473–490. https://doi.org/10.13109/prkk.2013.62.7.473

3. Kooij JJS (2012) Adult ADHD: Diagnostic assessment and treatment, 3rd ed. Springer, London

4. Ramos-Quiroga JA, Nasillo V, Richarte V, Corrales M, Palma F, Ibáñez P, Michelsen M, van de Glind G, Casas M, Kooij JJS (2019) Criteria and Concurrent Validity of DIVA 2.0: A Semi-Structured Diagnostic Interview for Adult ADHD. Journal of attention disorders 23(10):1126–1135. https://doi.org/10.1177/1087054716646451

5. Pettersson R, Söderström S, Nilsson KW (2018) Diagnosing ADHD in Adults: An Examination of the Discriminative Validity of Neuropsychological Tests and Diagnostic Assessment Instruments. Journal of attention disorders 22(11):1019–1031. https://doi.org/10.1177/1087054715618788

6. Kessler RC, Adler L, Ames M, Demler O, Faraone S, Hiripi E, Howes MJ, Jin R, Secnik K, Spencer T, Ustun TB, Walters EE (2005) The World Health Organization Adult ADHD Self-Report Scale (ASRS): a short screening scale for use in the general population. Psychological medicine 35(2):245–256. https://doi.org/10.1017/s0033291704002892

7. Wittchen HU, Zaudig M, Fydrich T (1997) SKID Strukturiertes Klinisches Interview für DSM-IV Achse I und II Handanweisung. Hogrefe, Göttingen

8. 8. Arbeitsgruppe Deutsche Child Behavior Checklist (1998) Elternfragebogen über das Verhalten von Kindern und Jugendlichen; deutsche Bearbeitung der Child Behavior Checklist (CBCL/4-18). Hogrefe, Göttingen

9. Döpfner M, Plück J, Kinnen C für die Arbeitsgruppe Deutsche Child Behavior Checklist (2014) Deutsche Schulalter-Formen der Child Behavior Checklist von Thomas M. Achenbach. Hogrefe, Göttingen10. Döpfner M, Plück J für die Arbeitsgruppe Deutsche Child Behavior Checklist (2014) Fragebögen zur Erfassung psychischer Probleme bei Erwachsenen. Deutschsprachige Fassung des Adult Self-Report for Ages 18-59 von Thomas M. Achenbach. Hogrefe, Göttingen

11. Petermann F (2012) Wechsler Adult Intelligence Scale - Fourth Edition. Deutschsprachige Adaptation der WAIS-IV von D. Wechsler. Pearson Assessment, Frankfurt am Main

12. Petermann F, Petermann U (2011) Wechsler Intelligence Scale for Children - Fourth Edition. Deutschsprachige Adaptation nach D. Wechsler. Pearson Assessment, Frankfurt am Main

13. Döpfner M, Görtz-Dorten A, Lehmkuhl G (2008) Diagnostik-System für psychische Störungen nach ICD-10 und DMS-IV für Kinder und Jugendliche. Hogrefe, Göttingen

14. Rösler M, Retz-Junginger P, Retz W, Stieglitz RD (2008) HASE - Homburger ADHS-Skalen für Erwachsene. Hogrefe, Göttingen

15. Döpfner M, Plück J, Kinnen C (2014) Deutsche Schulalter-Formen der Child Behavior Checklist von Thomas M. Achenbach. Hogrefe, Göttingen

16. Rush AJ, Gullion CM, Basco MR, Jarrett RB, Trivedi MH (1996) The Inventory of Depressive Symptomatology (IDS): psychometric properties. Psychological medicine 26(3):477–486. https://doi.org/10.1017/s0033291700035558

17. Drieling T, Schärer LO, Langosch JM (2007) The Inventory of Depressive Symptomatology: German translation and psychometric validation. International journal of methods in psychiatric research 16(4):230–236. https://doi.org/10.1002/mpr.226

18. Beck AT, Steer RA, Brown GK (1996) Manual for the Beck depression inventory-II. Psychological Corporation, San Antonio, TX

19. Kühner C, Bürger C, Keller F, Hautzinger M (2007) Reliabilität und Validität des revidierten Beck-Depressionsinventars (BDI-II). Befunde aus deutschsprachigen Stichproben (Reliability and validity of the Revised Beck Depression Inventory (BDI-II). Results from German samples). Der Nervenarzt 78(6):651–656. https://doi.org/10.1007/s00115-006-2098-7

20. Garnefski N, Kraaij V, Spinhoven P (2001) Negative life events, cognitive emotion regulation and emotional problems. Personality and individual differences(30):1311–1327

21. Garnefski N, Legerstee J, Kraaij VV, van den Kommer T, Teerds J (2002) Cognitive coping strategies and symptoms of depression and anxiety: a comparison between adolescents and adults. Journal of adolescence 25(6):603–611. https://doi.org/10.1006/jado.2002.0507

22. Garnefski N, Kraaij V (2007) The Cognitive Emotion Regulation Questionnaire. European Journal of Psychological Assessment 23(3):141–149. https://doi.org/10.1027/1015-5759.23.3.141

23. Loch N, Hiller W, Witthöft, M (2011) Der Cognitive Emotion Regulation Questionnaire (CERQ). Erste teststatistische Überprüfung einer deutschen Adaption. Zeitschrift für Klinische Psychologie und Psychotherapie(40 (2))

24. Wilcox RR, Schönbrodt F (2017) A Package of R. R. Wilcox’ Robust Statistics Functions. R package version 0.34

25. R Core Team (2019) R: A Language and Environment for Statistical Computing., Vienna, Austria
